# Quantum dot lasing from a waterproof and stretchable polymer film

**DOI:** 10.1038/s41377-022-00960-z

**Published:** 2022-09-15

**Authors:** Mohammad Mohammadimasoudi, Pieter Geiregat, Frederik Van Acker, Jeroen Beeckman, Zeger Hens, Tangi Aubert, Kristiaan Neyts

**Affiliations:** 1 Nano-Bio-Photonics Lab, Faculty of New Sciences and Technologies, University of Tehran, Tehran, Iran; 2 Liquid Crystals and Photonics Group, ELIS Department, Ghent University, Technologiepark-Zwijnaarde 126, 9052 Zwijnaarde, Belgium; 3 Physics and Chemistry of Nanostructures, Department of Chemistry, Ghent University, Ghent, Belgium; 4 Center for Nano- and Biophotonics (NB-Photonics), Ghent University, Technologiepark-Zwijnaarde 126, 9052 Zwijnaarde, Belgium

**Keywords:** Semiconductor lasers, Solid-state lasers

## Abstract

Colloidal quantum dots (QDs) are excellent optical gain materials that combine high material gain, a strong absorption of pump light, stability under strong light exposure and a suitability for solution-based processing. The integration of QDs in laser cavities that fully exploit the potential of these emerging optical materials remains, however, a challenge. In this work, we report on a vertical cavity surface emitting laser, which consists of a thin film of QDs embedded between two layers of polymerized chiral liquid crystal. Forward directed, circularly polarized defect mode lasing under nanosecond-pulsed excitation is demonstrated within the photonic band gap of the chiral liquid crystal. Stable and long-term narrow-linewidth lasing of an exfoliated free-standing, flexible film under water is obtained at room temperature. Moreover, we show that the lasing wavelength of this flexible cavity shifts under influence of pressure, strain or temperature. As such, the combination of solution processable and stable inorganic QDs with high chiral liquid crystal reflectivity and effective polymer encapsulation leads to a flexible device with long operational lifetime, that can be immersed in different protic solvents to act as a sensor.

## Abstract (web summary)

A quantum dot layer between two chiral liquid crystal mirrors forms a flexible foil that can emit laser light, also when immersed in water.

## Graphical abstract

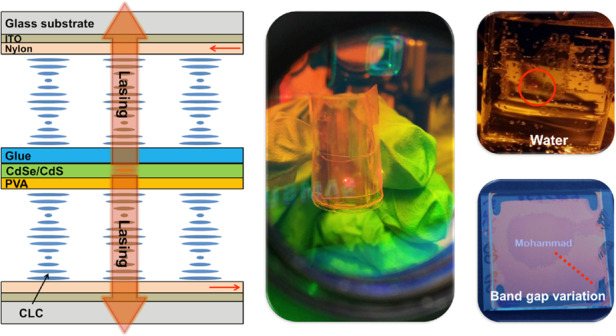

## Introduction

Over the last 10 years, various studies have shown the potential of colloidal semiconductor nanocrystals or quantum dots (QDs) as gain material for optically pumped lasers^1–3^. While initial devices were pumped by femtosecond pulsed lasers, quasi continuous-wave operation has been demonstrated and the first steps towards electrically pumped systems have been made^4,5^. The wide range of cavities used to make QD lasers—ranging from droplets and spheres to highly engineered disc resonators or distributed feedback structures—highlight the great design versatility that results from a high material gain and a suitability for solution-based processing^1,6^. QD-based gain layers exhibiting a material gain of 1000 cm^−1^ or more can be readily fabricated by dropcasting or spincoating, deposition techniques that can be combined with various material platforms^7^. These properties appear ideal for fabricating bendable and stretchable lasers in which a QD film provides optical gain, instead of organic dyes. However, while such a membrane laser could be a central building block for flexible opto-electronics^8–10^, only few studies have reported QD lasers on bendable films. Typically, these involve distributed feedback lasers made by depositing a QD film on a polymer with an imprinted surface grating^11,12^. Optical feedback and lasing in such structures strongly depend on the index contrast with the environment and on the size of the pump spot; in addition, the laser beams resulting from a surface grating typically have an asymmetric beam profile^13^.

A liquid crystal (LC) is an anisotropic state of matter arising from the orientational order of molecules in a liquid, that is used to realize tunable wavelength filters^14^ or tunable laser cavities^15^. LCs are promising for fabricating photonic components, given the design versatility of LC molecules, the relative ease of synthesis, and the electrical and optical tunability. Of particular interest are chiral nematic liquid crystals (CLCs), which exhibit self-ordering in a helical structure with a periodic modulation of the optical properties along the helical axis. As a result, circularly polarized light with the same handedness as the CLC is selectively reflected when the wavelength matches the one-dimensional photonic band gap (PBG) of the CLC. In this way, the helical structure acts as a self-organizing liquid mirror, the central wavelength of which is determined by the concentration of a chiral dopant in the mixture. Reactive liquid crystals make it possible to fix a LC configuration by polymerization, transforming the mechanical properties of the liquid into those of a polymer^16,17^. Cholesteric liquid crystal lasers^18^ and cholesteric polymer lasers emitting in the direction perpendicular to the substrate have been realized with light emitting dyes and with semiconductor quantum dots^19^.

By combining our expertise on QDs^2,6,20–23^ and LCs^15,17,24^ we have developed a free-standing thin film laser, consisting of a 100 nm thick layer of CdSe/CdS QDs sandwiched between two CLC mirrors of each about 7 μm. These mirrors form a cavity with a photonic band gap that overlaps with the photoluminescence spectrum of the QDs. We show lasing action under nanosecond optical pumping at 532 nm, where the laser wavelength can be tuned by simple adaptations of the cavity structure. The resulting laser light is circularly polarized, has a spatially narrow far field profile and a linewidth of 80 pm. Moreover, the QDCLC film is flexible, stretchable and waterproof. Lasing operation is possible while the film is exposed to polar protic solvents, such as formic acid, isopropanol, ethanol and water. Interestingly, the lasing wavelength is influenced by strain and temperature in a predictable manner, but not by the optical properties of surrounding media. Such a thin flexible laser with long lifetime (>10^7^ pulses) can be used as a temperature, pressure or strain sensor in water or in a biological solution.

## Results

**Fig. 1:**
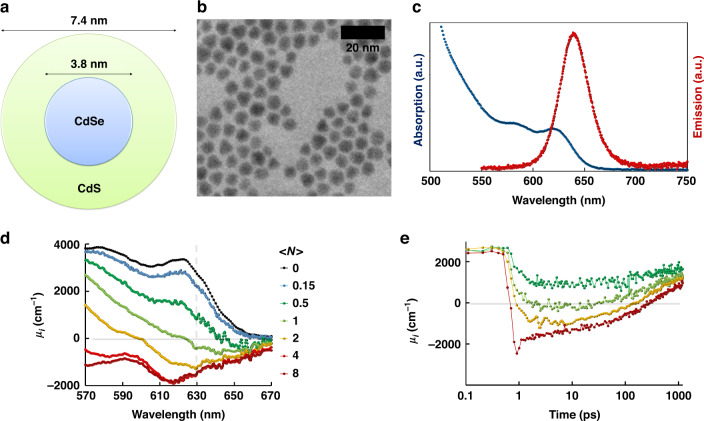
Properties of the synthesized QDs. **a** Schematic structure of a core/shell CdSe/CdS QD. **b** TEM image. **c** Absorption spectrum (blue) and photoluminescence spectrum (red) of the QDs dispersed in toluene. **d** QD absorption (μ_i_ > 0) or gain (μ_i_ < 0) spectrum as a function of the excitation level <*N* > at 3 ps after the pulse excitation. **e** Corresponding time-dependency of the gain spectrum at a fixed probe wavelength of 630 nm

The CdSe/CdS QDs are manufactured following the procedure described in the Methods (section QD fabrication). First, CdSe core QDs, with a wurtzite structure and an average diameter of 3.8 nm, are synthesized^25^. On these core QDs, a CdS shell is grown to obtain CdSe/CdS QDs with an average diameter of 7.4 nm. Previous work has shown that such large core/shell QDs are best suited for optical gain, offering the best compromise between gain threshold, lifetime and magnitude^20^. Figure 1a, b show the structure of the QD and a transmission electron microscopy (TEM) image of the QDs respectively. The absorption and emission spectra of the QDs are measured in toluene and are shown in Fig. 1c. The QDs have an emission spectrum centered around 640 nm and can be efficiently excited with blue or UV light. To quantify the optical gain characteristics of these core/shell QDs, we use transient absorption spectroscopy after photo-excitation of a colloidal dispersion of QDs in toluene by a 530 nm light pulse. A broadband probe pulse measures the absorbance of the sample, where negative values indicate net optical gain. In Fig. 1d the spectral intrinsic absorption coefficient *μ*_*i*_ is shown, 3 ps after photoexcitation. After correction for the field factor and the quantity needs to be diluted to obtain the modal absorption or gain by using the modal confinement and volume fraction of the QD layer. At increasing pump fluence, expressed by the average number of excitations per QD < *N* > , a transition from net absorption to net stimulated emission, *μ*_*i*_ < 0, was observed. The maximum intrinsic gain near 630 nm is close to 1500 cm^−1^. Taking into account the local field factor, this yields a material gain of about 3600 cm^−1^. Figure 1e shows the dynamics at 630 nm of the gain, showing that net optical gain persists for several hundred ps, in-line with state-of-the-art CdSe/CdS QDs^20^. The raw transient absorption data is represented in Supplementary Fig. S1.

**Fig. 2:**
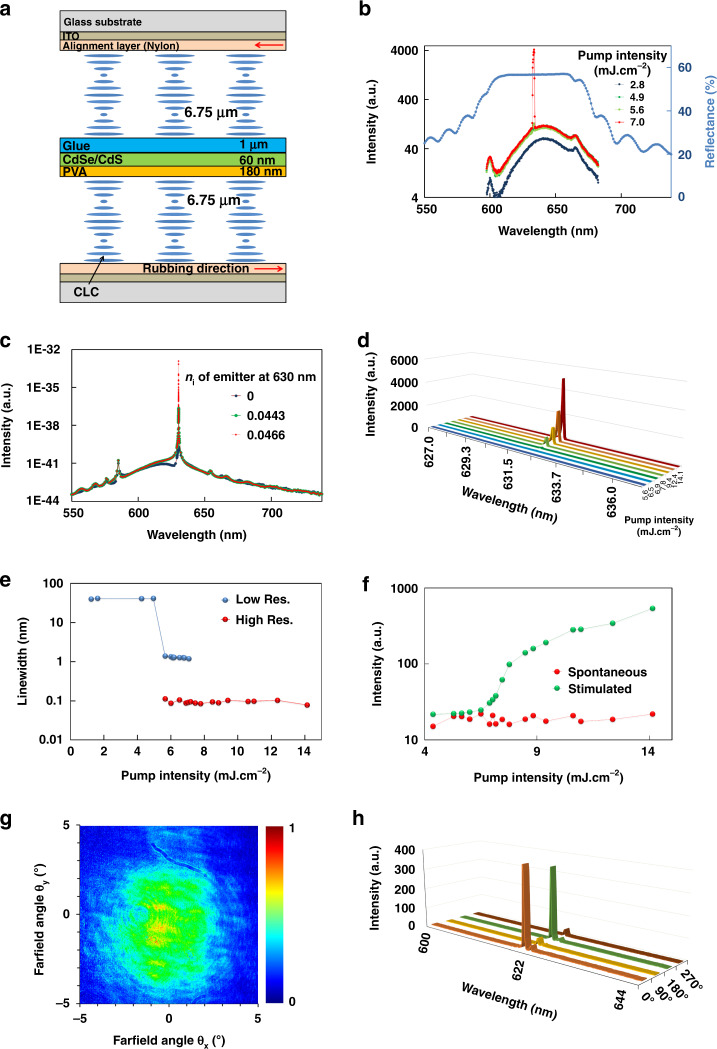
QDCLC laser structure and characteristics. **a** Schematic structure of the QDCLC laser. **b** Measured reflection spectrum of the QDCLC layer stack (light blue) and emission spectra for various pump intensities. **c** Simulated amplified spontaneous emission spectra for various imaginary parts of refractive index of the QD layer, representing various gain coefficients. **d** Measured high resolution spectra of the QDCLC emission for various pump intensities. **e** Integrated intensity of the lasing peak (around 632 nm) and of the spontaneous emission (around 650 nm) as a function of the pump intensity. **f** Linewidth of the measured laser beam, measured with the low (blue dots, as in **b**) and high (red dots, as in **d**, **e**) resolution setup, as a function of pump intensity. **g** Measured intensity of the QDCLC emission as a function of the emission angle. **h** Integrated emission of the laser peak after transmission through a quarter wave plate and a rotating polarizer

For light incident along the helical axis of a CLC, the width of the PBG is Δ*λ* = Δ*np* with *Δn* = *n*_e_ *−* *n*_o_ the birefringence of the LC, and *P* the pitch, the distance over which the director rotates 360 °. The CLC mirror reflects circularly polarized light matching the handedness of the helix, while circularly polarized light with the opposite handedness propagates unhindered (Supplementary Fig. S2)^21,26^. Solid CLC layers are made by photo-polymerization of a liquid crystalline acrylate mixture to which an appropriate amount of chiral dopant has been added. More details can be found in the Methods section, CLC mirror fabrication. The resulting CLC thin film mirror remains stable up to 100 °C and can be separated from the substrate. Compared to previously reported CLCs, we use an adapted method that makes it possible to realize CLC mirrors with large domains, in the order of 10 mm^2^ ^27–29^. Two polymerized CLC mirrors are deposited on separate substrates and between them a stack of three layers is realized: a layer of PVA (280 nm), a layer of QDs (100 nm) and a layer of glue (940 nm) as explained in the Methods section on laser fabrication. The full structure of the CLC laser between glass substrates is schematically shown in Fig. 2a. Figure 2b shows the reflection spectrum of the resulting QDCLC stack between glass substrates for unpolarized light, indicating a PBG between 600 nm and 660 nm, which covers most of the PL emission spectrum of the QDs. The QDCLC laser is pumped by a nanosecond laser beam (532 nm) in a measurement set up that is explained in more detail in the Methods section. The diameter of the pump laser beam is 200 µm as shown in Supplementary Fig. S4. The emission spectra of the QDCLC layer below and above the threshold in Fig. 2b clearly indicate the onset of lasing in the defect mode of the multilayer stack. Figure 2c shows numerically simulated emission spectra in the perpendicular direction, based on the measured emission spectrum in solution, including optical gain implemented by adding an imaginary part to the refractive index of the QD layer. The imaginary part of the refractive index at 630 nm is varied between 0 (no gain) and 0.0466 (for gain just below the threshold for lasing). The isotropic material between the CLC layers leads to an optical defect mode, that results in lasing at 630 nm for sufficiently strong pumping. The defect mode has losses related to reflections, in particular at the interfaces of the QD layer which has the highest index of refraction. There are also band edge modes (at 585 nm and 655 nm) that are associated with the pitch of the reflection band of the CLC layers, which match the measurement results quite well.

Figure 2d illustrates the measured emission spectrum of the QDCLC layer for various pump intensities, measured with a resolution of 0.03 nm. The integrated intensity of the lasing peak and the intensity integrated in a wavelength region with spontaneous emission are plotted in Fig. 2e for various pump intensities, revealing a typical S-shaped curve indicative of lasing (see also Supplementary Fig. S5 for a graph on a linear scale). From this figure we obtain the pump threshold for lasing of about 7 mJ.cm^−2^. The full width at half maximum (FWHM) of the emission spectrum as a function of the pump power, measured with the low (blue dots) and high (red dots) resolution setup, is provided in Fig. 2f. The results indicate an abrupt decrease of the spectral FWHM to 0.08 nm at the pump threshold (7 mJ.cm^−2^), which is close to the limit of the measurement set up, and may therefore be over-estimated. This value is smaller than the FWHM value found in previous reports^30^. The far field emission in Fig. 2g shows the spatial coherence and angle dependency of the symmetric laser emission. The circular polarization state of the emitted laser beam is verified by placing a quarter waveplate oriented at 45° and a linear polarizer in front of the fiber probe (See Fig. S6a in Supplementary). The emission spectra of the QDCLC layer for the polarizer oriented at 0°, 90°, 180° and 270° are plotted in Fig. 2h. The integrated intensity of the lasing peak as a function of the orientation angle of the polarizer is plotted in Supplementary Fig. S6b. The fact that the emission is close to zero when the polarizer is oriented at 90 ° indicates the laser emission is circularly polarized, as expected for a defect-mode CLC laser^31^.

**Fig. 3:**
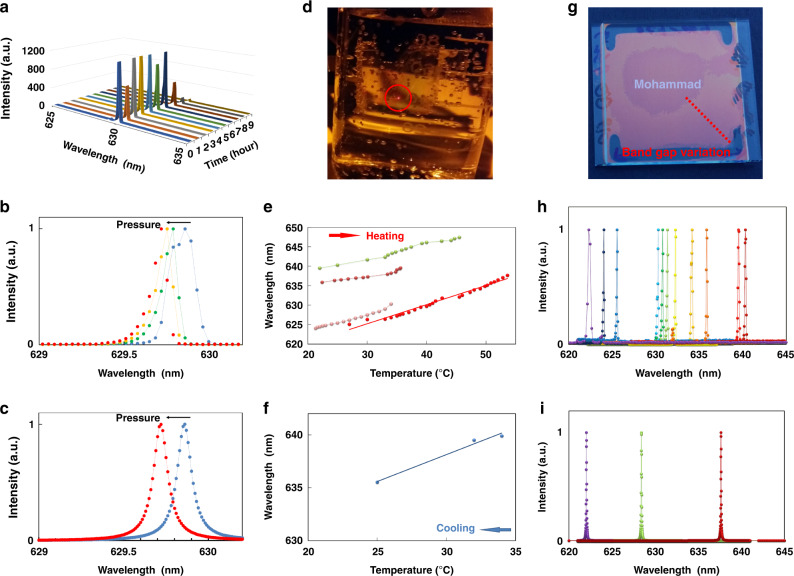
Experiments evaluating external factors on the QDCLC film. **a** Lasing spectra of a free-standing film QDCLC laser for 9 h (32 million pulses) of continuous operation at 1 kHz. **b** Measured (four spectra, each time with a clamp added) and **c** simulated (for LC with reduced pitch) lasing spectra as a function of the pressure exerted on the QDCLC stack between two glass plates. **d** Photograph of the free standing QDCLC laser in hot water (54 °C). **e** Red shift (for four locations on the layer) and **f** blue shift of the lasing wavelength by heating and cooling of the water. **g** Photograph of a CLC mirror between two glass plates with two domains, in which the variation in the thickness and in the reflection band is observed along the red dashed line. **h** Measured (for 11 different locations) and **i** simulated (for three different CLC pitches) lasing spectra for a free-standing film with variable CLC pitch

A second series of experiments focuses on the influence of external factors on the properties of the QDCLC films. To achieve free-standing films, the two glass substrates are carefully detached from the stack, and a film with total thickness around 15 µm is obtained. The lasing stability of a free-standing QDCLC film has been investigated by continuously recording lasing spectra during 9 h, equivalent to more than 32 million pulses, using a pump intensity of 8 mJ.cm^−2^ and a frequency of 1 kHz. The high-resolution spectra as a function of time are shown in Fig. 3a and the variation of the linewidth is shown in Fig. S7a. After keeping the QDCLC laser on the shelf for one year the device still works properly. The influence of pressure on the laser emission of the QDCLC film has been measured by placing clamps on the left and right side of the film between two glass substrates. Figure 3b shows the spectra of the QDCLC laser emission for different pressures exerted by the clamps. When the pressure is increased a continuous blue shift of the laser peak is experienced. Figure 3c shows the simulated laser spectra for an unstrained QDCLC layer and for a QDCLC layer in which all layers in the stack have been reduced in thickness, corresponding to a strain of ɛ = −2.2 × 10^−4^, and a reduction of the total stack thickness between the glass plates of 3.3 nm.

The free-standing QDCLC film laser is highly resistant to water. This has been verified by placing the QDCLC film inside a beaker of water and measuring the laser emission under water. The emission spectrum of the laser under water is shown in Fig. S7b. Essentially, the obtained spectrum and threshold are unchanged. A simple way to control the temperature of the laser and minimize heating by the pump laser, is by measuring the QDCLC free-standing film under water. By heating the water in the beaker, the effect of the temperature on the emission spectrum can be measured, however at higher temperatures the measurement is disturbed by bubbles that are created on the surface of the beaker (Fig. 3d), and in that case the heating/cooling cycle was terminated. The wavelength of the laser emission shifts to the red when the temperature increases and shifts back to the blue by cooling as shown in Fig. 3e, f respectively for different samples. Fitted lines indicate a 0.5 nm shift of the lasing spectra per °C. Lasing of the QDCLC film in water can be observed up to 54 °C. The linewidth of the measured laser beam is almost independent of the temperature (see Fig. S8 in Supplementary).

Figure 3g shows a photograph of a single CLC mirror between two glass substrates with the dimension of edge equal to one inch, containing two domains. In the central region of the device the CLC is slightly thinner than in the outer region and therefore the wavelength of the reflection is blue-shifted. The discrete change in color is due to a defect line that is associated with a reduction of the twist by 180° in the inner region, compared to the outer region. In the outer region (along the red dashed line in Fig. 3g) the reflection band shifts continuously, due to a variation in thickness and pitch, while the total twist remains the same. When the emission spectrum of a free-standing film is measured across a region with variable CLC pitch, Fig. 3h is obtained, which illustrates the spatial tunability of the QDCLC laser over a wavelength range of 18 nm^32^. The effect of the variation of the thickness of the CLC layer (and the pitch of the CLC) on the laser emission spectrum is simulated numerically (Fig. 3i). A good correspondence with the measurements is obtained by using a CLC with 17 full rotations of the director and with a pitch that varies between 391 nm and 403 nm (that is 6 nm shorter or 6 nm longer than in the middle of the region).

**Fig. 4:**
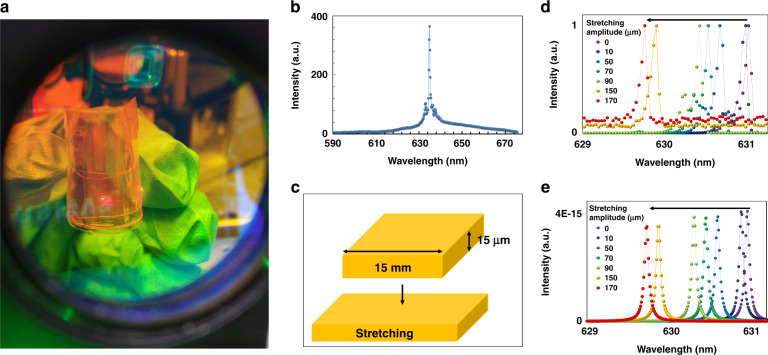
Lasing emission of bend and stretched QDCLC films. **a** Photograph and **b** Emission spectrum of a bent QDCLC laser on an adhesive film. **c** Illustration of how the free-standing QDCLC film with a thickness of 15 µm and a length of 15 mm is stretched to increase the length by 170 µm. **d** Emission spectra of the stretched laser with various stretch amplitudes and **e** corresponding simulation in which the thickness of the layers is reduced proportionally with the stretching amplitude

To investigate bending of the QDCLC laser, the free-standing film is attached to an adhesive tape. Figure 4a demonstrates lasing of the bent QDCLC film attached onto an adhesive tape (a similar picture is shown in Supplementary Fig. S9a). The emission spectrum of the bent laser is plotted in Fig. 4b. Figure S9b (Supplementary) shows a photograph of the QDCLC film on an adhesive tape that illustrates that the device has low scattering (due to the monodomain structure) and relatively high transmission (except for wavelengths within the photonic band gap).

By stretching the free-standing QDCLC film (Fig. 4c), the lasing wavelength shifts to the blue side of the spectrum (Fig. 4d), proportionally with the magnitude of the strain. The linewidth of the laser emission remains practically constant during stretching of the film (Fig. S10a). The maximal strain in the direction of stretching is 170 µm/15 mm = 11 × 10^−3^. Simulations (Fig. 4e) indicate that a decrease in the thickness of the QDCLC laser leads to a blue shift of the lasing wavelength. From the magnitude of the blue shift of the spectrum, we can estimate the strain deformation perpendicular to the film as 1.26 nm/630 nm = −2 × 10^−3^. By taking the ratio of the lateral over the longitudinal strain, Poisson’s ratio ν can be calculated as 0.18. Stretching of the laser is reversible with the wavelength shift for increasing and decreasing shift shown in Fig. S10b.

## Conclusions

In this work we have demonstrated that by embedding a thin layer of semiconductor QDs between two polymerized CLC mirrors, stable lasing can be obtained with a low power threshold for excitation with a 532 nm pulsed ns laser. The pump threshold is in the order of 7 mJ.cm^−2^, the lasing wavelength is around 630 nm and the emission is circularly polarized. The QDCLC film can be detached from the substrates to obtain a free-standing film. Lasing of this film can be observed for several hours, and in water that is heated to above 50 °C. The wavelength of the lasing can be modified by pressure on the glass plates, by stretching the free-standing film, by changing the temperature or by simply shifting the pump beam to an area in which the CLC has a slightly different pitch.

The unique combination of inorganic QDs that provide stable long-term optical gain, with polymerized CLC that provides a high reflectivity, allows to realize a thin, flexible and at the same time stable optically pumped laser. This approach could be applied to realize cheap large area devices that can be used in laser-based sensors or tunable lasers.

## Methods

### Materials

CdO (≥99.99%), oleyl alcohol (85%) was obtained from Sigma-Aldrich. N-octadecylphosphonic acid (≥97%) and N-tetradecylphosphonic acid (≥97%) from PlasmaChem GmbH. Trioctylphosphine (TOP, ≥97%) and sulfur (99.999%) were purchased from Strem Chemicals. Trioctylphosphine oxide was purchased from Merck Millipore. Selenium (200 mesh, 99.999%) and oleic acid (90%) were obtained from Alfa Aesar. The reaction solutions of TOP-Se (2 M) and TOP-S (2.4 M) were prepared by dissolving 1.56 g of the selenium powder and 0.77 g of sulfur in 10 mL of TOP.

### CdSe/CdS QD fabrication

CdSe/CdS core/shell quantum dots have been produced by previously reported procedures^25,33,34^, involing the preparation of the CdSe (core) QDs and growth of a CdS shell. CdSe wurtzite particles were synthesized based on CdO, n-tetradecylphosphonic acid (TDPA), oleyl alcohol (OlOH) and 10 g trioctylphosphine oxide (TOPO), with 1:6:16 as molar ratio of Cd:TDPA:OlOH. The reaction mixture was heated for 1 h at 150 °C under nitrogen atmosphere and heated to 350 °C to dissolve CdO, followed by injections of 2 mL of TOP and a 2 M TOP-Se solution, the latter having a Cd:Se molar ratio of 1:2. The reaction time is limited to a few seconds by reducing the temperature. The QDs were precipitated from 20 ml of methanol and collected by centrifugation at 4000 rpm for 3 min. The QDs were finally purified with toluene and methanol.

The concentration of the wurtzite QDs dispersion in toluene was estimated by comparing the absorbance of the mixture with the intrinsic absorption coefficient based on effective medium theory^22,35^. The size of the CdSe particles was estimated from the position of the first excitonic absorption peak, based on the sizing curve of Jasieniak et al.^23^, while the average diameter of the QDs was estimated from TEM images as 7.6 nm. The quantum efficiency of the QDs solution was obtained from a two-measurement approach^36^, and estimated as 64%. With a Cs corrected JEOL 2200 FS microscope, bright-field transmission electron microscopy (TEM) images were obtained. A Perkin Elmer Lambda 950 spectrometer was used for the absorption spectra.

### Transient absorption spectroscopy

To excite the samples, 110 fs pump pulses, centered at 530 nm, were used. The pulses were generated from the 800 nm fundamental (Spitfire Ace, Spectra Physics) through non-linear conversion in an OPA (Light Conversion TOPAS). A delay stage was used to delay the pulses relative to the pump. The probe spectrum covers the range from 350 nm to 750 nm. The quantum dots were dispersed in toluene and stirred to avoid charging or photo-degradation. The average number of absorbed photons <N > at time zero, is estimated from the photon flux, the length of the cuvette L and the absorption cross-section at the pump wavelength. The photon flux is estimated from the beam area *A* = 2*π*σ_*x*_σ_*y*_, estimated with a Thorlabs CCD beam profiler, with *σ* the standard deviations in the *x,y* directions.

### CLC mirror fabrication

The polymerizable CLC mixture was obtained by adding two liquid crystalline diacrylate monomers (RM257, 40 wt% and RM82, 21 wt%, Merck) to 32 wt% liquid crystalline monoacrylate monomers (RM105, Merck). The ordinary and extraordinary refractive indices for RM257 are *n*_o_ = 1.508, *n*_e_ = 1.687, and the melting and clearing points are at 66 °C and 127 °C, respectively. RM82 and RM105 are added to increase the nematic range. The photo-initiator (Irgacure 819, 3 wt%, BASF) and the polymerization inhibitor (tert-Butylhydroquinone, 4 wt%, Sigma-Aldrich) are added. The right-handed chiral dopant (BDH1305, Merck) yields the desired helical structure. The PBG of the mirrors is adjustable with the selection of the appropriate chiral dopant concentration. The molecular structures are shown in Supplementary, Fig. S3.

A magnetic stirrer is used to mix the materials. Two standard glass substrates with 20 nm thick conductive Indium-Tin-Oxide (ITO) are used to fabricate cells. Nylon 66 is coated on the substrates with antiparallel rubbing to obtain a CLC with helical axis perpendicular to the glass substrates. Spacers balls with 6.75 µm diameter (Sekisui chemical) mixed inside the glue which is deposited along the edges of the substrate defines the gap between the substrates. The empty cell is filled with the polymerizable CLC mixture aided by the capillary effect in vacuum on a hot stage in the isotropic phase (92 °C). The cells are gradually cooled down to the extent that the liquid crystal orients itself into a helical structure, forming a uniform film with few domains. The polymerization of the CLC mixture is achieved by exposing the cell to one minute of UV light (30 mWcm^−2^) from a mercury lamp (with main emission around 365 nm, by using a filter which blocks both shorter and longer wavelengths).

### QDCLC fabrication

The upper glass of the polymerized CLC cell is delaminated by a cutter. Then a PVA coating with thickness approximately 180 nm is deposited onto the polymerized CLC layer to protect it against toluene in the following step. Colloidal QDs in toluene are coated onto the PVA layer to realize a 100 nm active layer, after evaporation of the toluene. A second polymerized CLC layer (of which the second glass substrate has been delaminated) is glued onto the active layer using low viscosity curable glue. By exerting a pressure between the two substrates during polymerization of the glue, the thickness of the glue is reduced to approximately 1 µm (Fig. 2a). The resulting glued QDCLC device has now two CLC mirrors and two glass substrates. The upper glass substrate of the QDCLC laser is again delaminated by a cutter. In order to obtain a free-standing film, the QDCLC film (consisting of two CLCs, the QD layer and the glue) is separated from the substrate by a cutter or an adhesive tape.

### Transmission measurement

The spectral transmittance of the QDCLC device between two glass plates is determined by using a spectrophotometer (Perkin Elmer).

### Lasing characterization

A Q-switched Nd:YAG laser is used to generate second harmonic laser pulses with wavelength 532 nm, with a pulse duration of 10 ns, and pulse repetition frequency 1 kHz. The power of the 532 nm pump beam is tuned by an attenuator and the beam is fixated on the sample using a lens (focal length 5 cm) with an angle of incidence of 45° with the normal of the QDCLC layers. The emission of the QDCLC laser is collected by a multimode fiber of 200 µm core diameter and 0.22 NA at a distance of 20 mm, along the normal of the QDCLC layers. The detectors are a Compact CCD Spectrometers (CCS200M, Thorlabs) and an imaging spectrometer with 300 mm focal length, 300 gmm^−1^ grating blazed at 510 nm and a 512 × 512 emCCD camera (Princeton Pro:EM 512). We used a Thorlabs CCD beam profiler for the far-field measurements.

### Simulation

The spontaneous emission and amplified spontaneous emission of the QDCLC stack in the perpendicular direction is simulated by an in-house developed software written in Matlab^24^. The calculation is based on spontaneous emission from isotropic electrical dipole emitters with distribution according to the intrinsic emission spectrum, and on a matrix method for transmission and reflection on homogeneous anisotropic slabs. The spectral dependent gain in the QD layer is represented by a complex imaginary part in the refractive index, which is assumed to be proportional to the intrinsic emission spectrum. The parameters for the layers are given in Table S1 (Supplementary).

## Supplementary information

Supplementary Information
