# Supplementary material for: Quantum dot lasing from a waterproof and stretchable polymer film
Source: Light Sci Appl. 2022 Sep 15;11:275. doi: 10.1038/s41377-022-00960-z (PMC9475037; doi:10.1038/s41377-022-00960-z)
Supplement: Supplementary file 1 — Supplementary Information [file 41377_2022_960_MOESM1_ESM.docx]

Supplementary Information for

**Quantum dot lasing from a waterproof and stretchable polymer film**

Mohammad Mohammadimasoudi^1,2^, Pieter Geiregat^3,4^, Frederik Van Acker^2,4^,Jeroen Beeckman^2,4^, Zeger Hens^3,4^, Tangi Aubert^3,4^, Kristiaan Neyts^2,4^

^1^ Nano-Bio-Photonics Lab, Faculty of New Sciences and Technologies, University of Tehran, Tehran, Iran

^2^ Liquid Crystals and Photonics Group, ELIS Department, Ghent University, Technologiepark-Zwijnaarde 126, 9052 Zwijnaarde, Belgium

^3^ Physics and Chemistry of Nanostructures, Department of Chemistry, Ghent University, Belgium

^4^ Center for Nano- and Biophotonics (NB-Photonics), Ghent University, Technologiepark-Zwijnaarde 126, 9052 Zwijnaarde, Belgium.


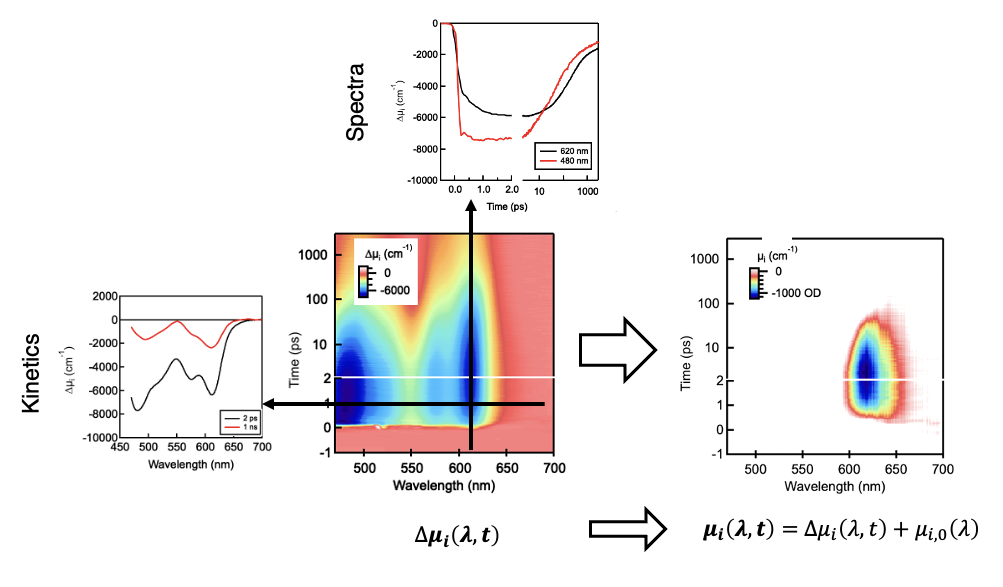


**Fig. S1** Raw transient absorption data obtained as 2D color maps, normalized to volume fraction and path length to obtain $\Delta\mu_{i}$. Summing this together with the linear absorption coefficient $\Delta\mu_{i,0}$, we obtain the time and wavelength dependent absorption coefficient $\mu_{i}$. On the right: region where this quantity turns negative making it in effect a “gain” map.


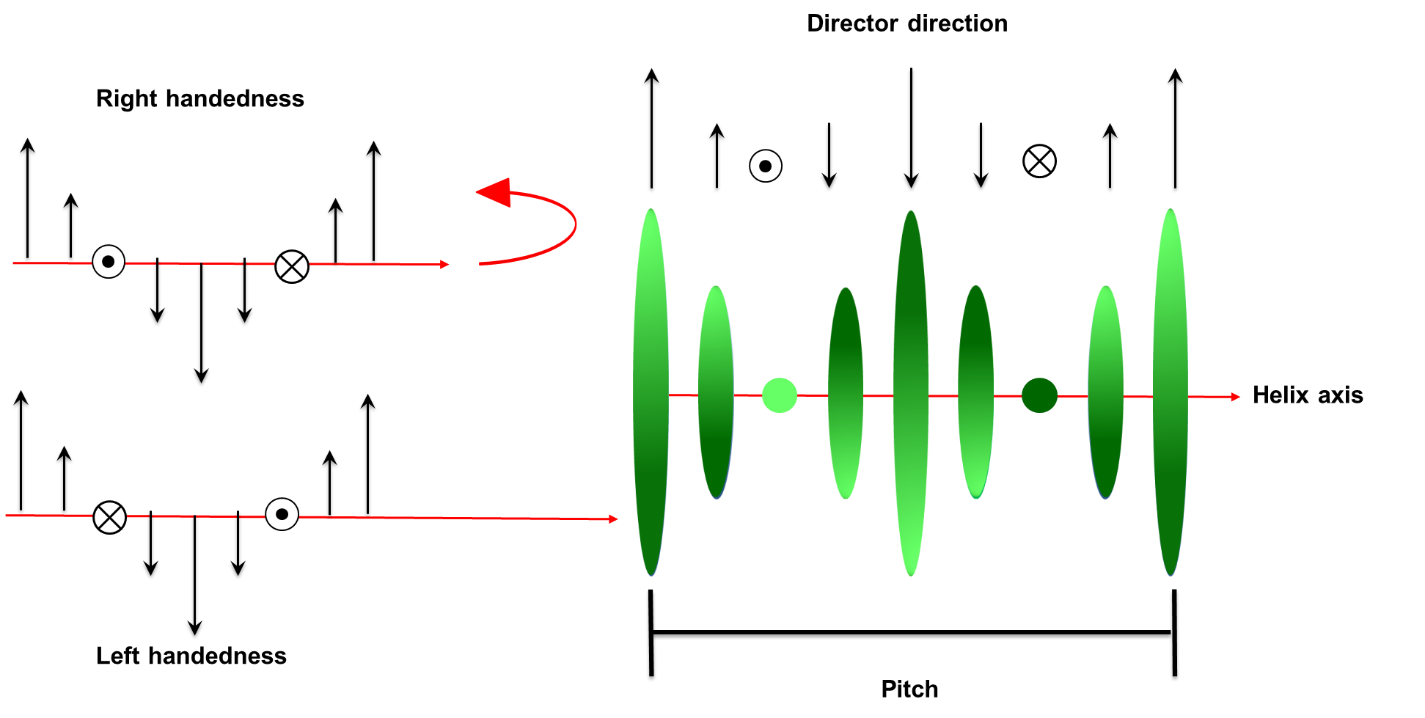


**Fig. S**2**.** Configuration of the local director for one pitch of the CLC. Circularly polarized light with the same handedness as the CLC is reflected with the same handedness.


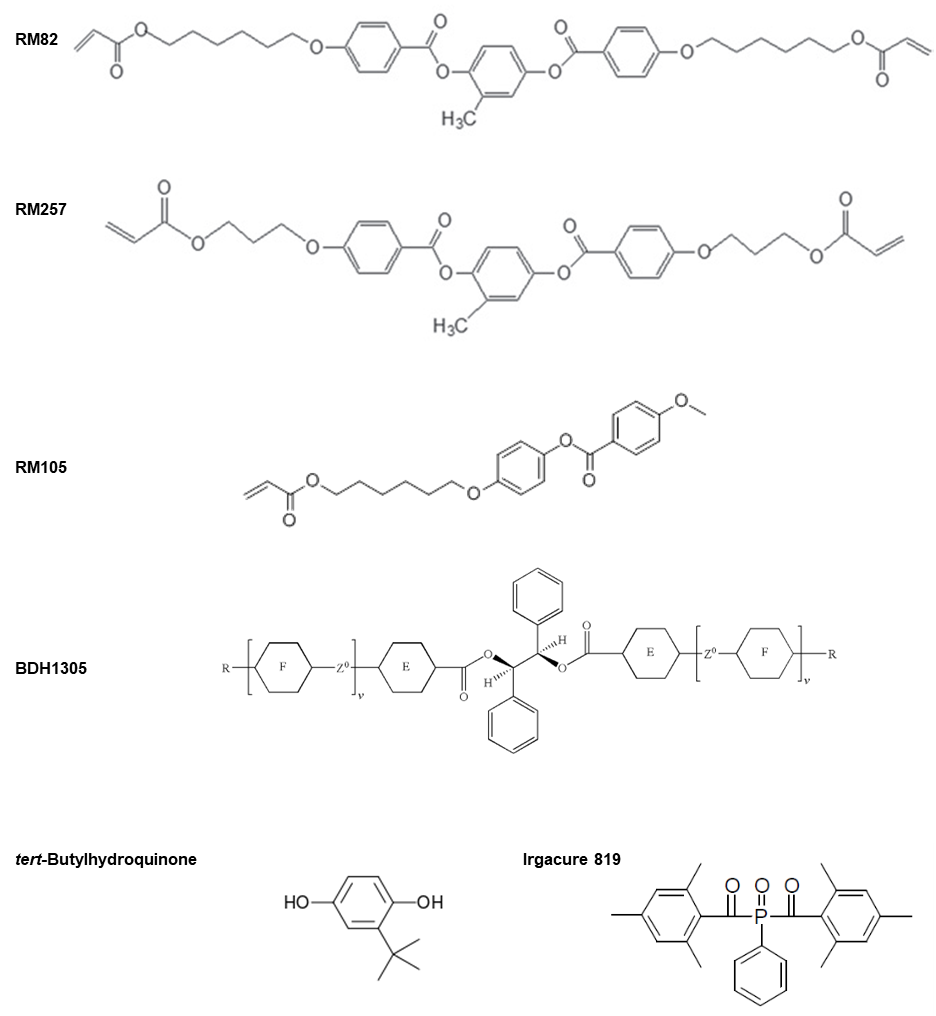


**Fig. S3.** Chemical structure of two liquid crystalline diacrylate monomers with side groups of different lengths (RM 82 and RM 257), liquid crystalline monoacrylate monomer (RM 105), chiral dopant (BDH 1305), inhibitor (tert-Butylhydroquinone) and initiator (Irgacure 819).


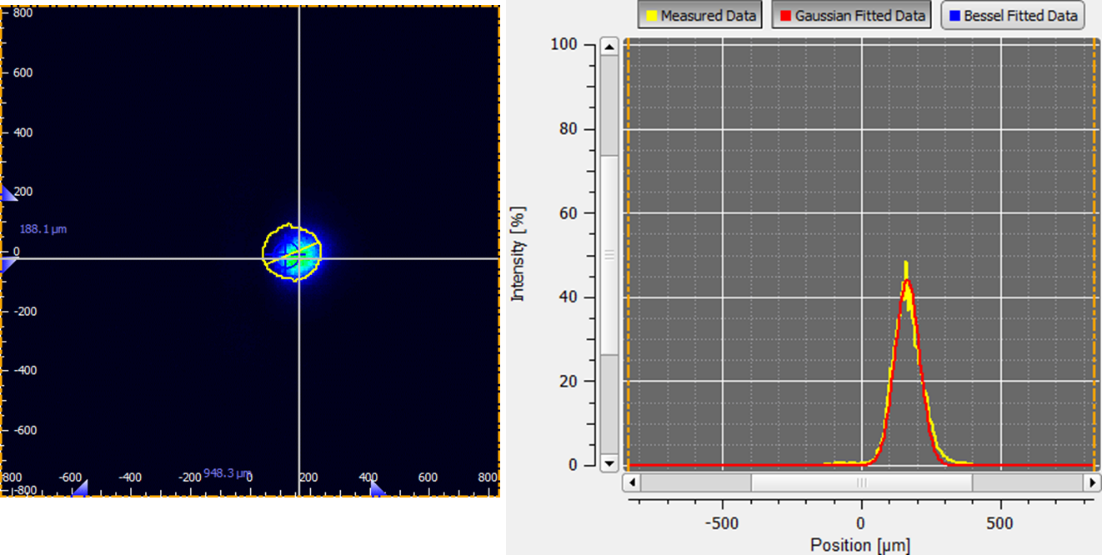


**Fig. S4.**  Measurement of the diameter of the nanosecond 532 nm pump laser beam (about 200 µm).

**Fig.** **S5**. Integrated intensity of the QDCLC laser emission laser as a function of the pump intensity, indicating a linear relation.


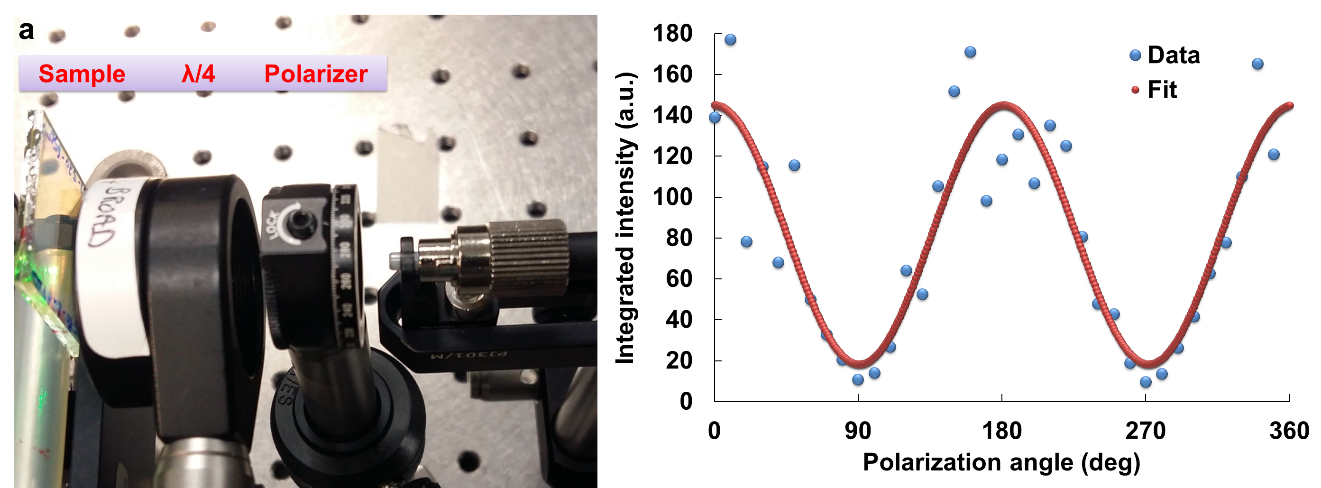


**Fig. S6.** **a** Photograph of the set up to investigate the polarization state of the QDCLC laser emission, with the slow axis of the quarter wave plate at an angle of 45 degrees. **b** Integrated intensity of the QDCLC laser emission after transmission through the quarter wave plate and the polarizer, as a function of the angle of the polarizer. The low transmission when the polarizer is oriented at 90 degrees indicates that the QDCLC laser emission is to a high degree circularly polarized.


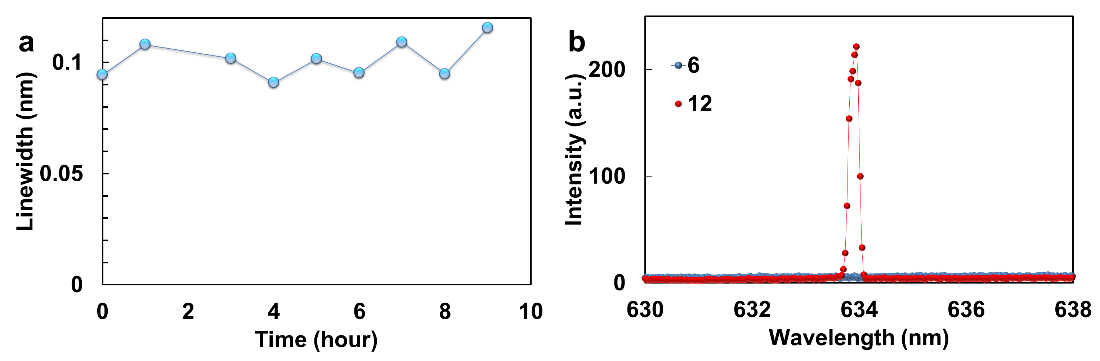


**Fig. S7** **a** Linewidth of the QDCLC laser beam recorded during 8 hours of continuous operation. **b** Emission spectra of the QDCLC laser immersed in water for excitation levels of 6 and 12 mJcm^-2^


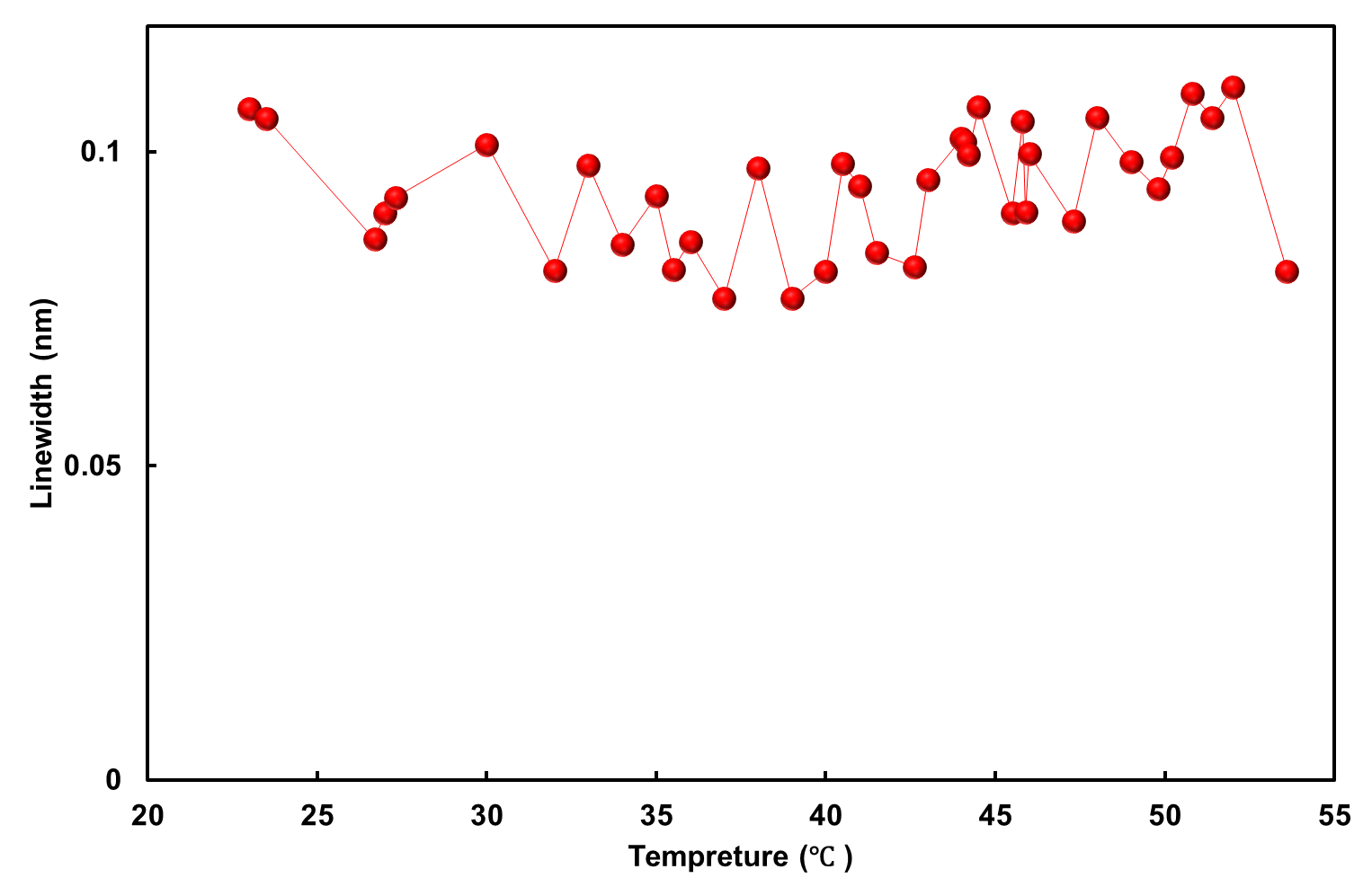


**Fig. S8** Linewidth of the QDCLC laser beam as a function of temperature.


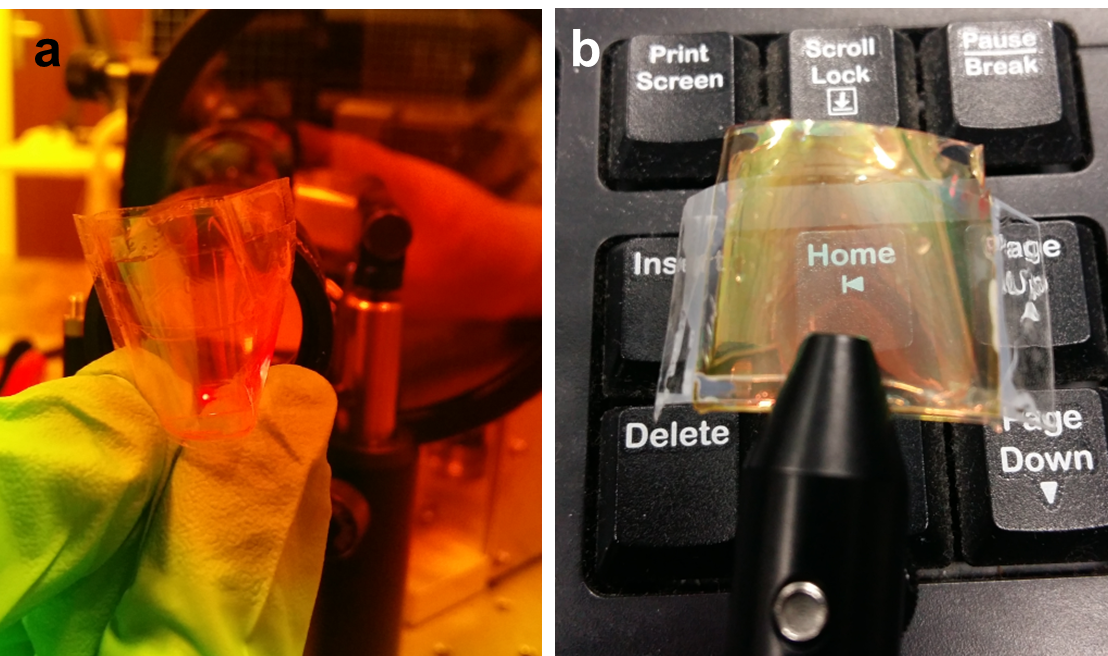


**Fig. S9** Photographs of a thin film QDCLC laser **a** bend and pumped with a green laser, with laser emission seen as a red spot (a notch filter is used between the laser and camera to block the pump laser). **b** bend and placed on a keyboard to illustrate the lack of scattering: the white “Home” key is clearly visible.


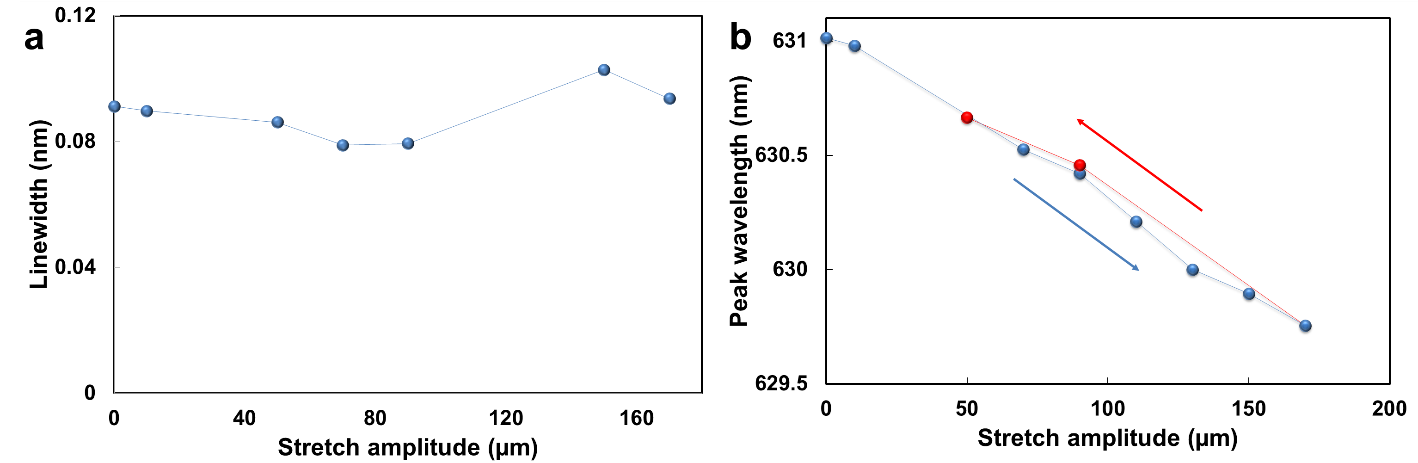


**Fig. S10** Influence of the stretching amplitude on the flexible QDCLC laser. **a** Linewidth of the QDCLC laser beam. **b** Peak wavelength of the lasing spectrum with the blue dots indicating increasing stretching and the red dots indicating decreasing stretching. This indicates the reversibility of the stretching.

**Table S1** Layer properties used for the numerical simulation of the emission spectrum.

| material | thickness (nm) | index n_o_ | index n_e_ | pitch (nm) |
| --- | --- | --- | --- | --- |
| glass |  | 1,515 |  |  |
| CLC | 6930 | 1,508 | 1,687 | 385 |
| PVA | 278,5 | 1,484 |  |  |
| QDs | 100 | 2,050 |  |  |
| glue | 947,5 | 1,560 |  |  |
| CLC | 6930 | 1,508 | 1,687 | 385 |
| glass |  | 1,515 |  |  |

The emission is calculated in the perpendicular direction, taking into account reflections in the CLC and at the interfaces between layers. The CLC has a thickness corresponding to 18 pitches of 385 nm. The CLC is birefringent with two refractive indices. The layer of QDs contains also ligands and therefore the refractive index in the table is estimated to be lower than that of the pure QDs. The thickness of the glass is set to infinity to eliminate reflections from the glass/air surface. The simulation is based on the emission of an electrical dipole and takes into account interference effects due to partial reflections.
